# Electrically‐Shielded Coil‐Enabled Battery‐Free Wireless Sensing for Underwater Environmental Monitoring

**DOI:** 10.1002/advs.202414299

**Published:** 2025-01-31

**Authors:** Ke Wu, Xia Zhu, Stephan W. Anderson, Xin Zhang

**Affiliations:** ^1^ Department of Mechanical Engineering Boston University Boston MA 02215 USA; ^2^ Photonics Center Boston University Boston MA 02215 USA; ^3^ Chobanian & Avedisian School of Medicine Boston University Medical Campus Boston MA 02118 USA

**Keywords:** battery‐free, electrically‐shielded coil, near‐field antenna, underwater environmental monitoring, wireless sensing

## Abstract

Battery‐free wireless sensing in extreme environments, such as conductive solutions, is crucial for long‐term, maintenance‐free monitoring, eliminating the limitations of battery power and enhancing durability in hard‐to‐reach areas. However, in such environments, the efficiency of wireless power transfer via radio frequecny (RF) energy harvesting is heavily compromised by signal attenuation and environmental interference, which degrade antenna quality factors and detune resonance frequencies. These limitations create substantial challenges in wirelessly powering miniaturized sensor nodes for underwater environmental monitoring. To overcome these challenges, electrically‐shielded coils with coaxially aligned dual‐layer conductors are introduced that confine the electric field within the coil's inner capacitance. This configuration mitigates electric field interaction with the surrounding medium, making the coils ideal for use as near‐field antennas in aquatic applications. Leveraging these electrically‐shielded coils, a metamaterial‐enhanced reader antenna was developed and a 3‐axis sensor antenna for an near‐field communication (NFC)‐based system. The system demonstrated improved spectral stability, preserving resonance frequency and maintaining a high‐quality factor. This advancement enabled the creation of a battery‐free wireless sensing platform for real‐time environmental monitoring in underwater environments, even in highly conductive saltwater with salinity levels of up to 3.5%.

## Graphical abstract

This study develops a battery‐free wireless sensing platform for underwater monitoring in conductive solutions. By introducing electrically‐shielded coils with coaxially aligned dual‐layer conductors, electric field interference is mitigated, enhancing the spectral stability of near‐field antennas. Integrated with a metamaterial‐enhanced reader antenna and a 3‐axis sensor antenna, the near‐field communication (NFC)‐based system enables real‐time environmental monitoring in saltwater with up to 3.5% salinity.

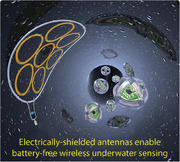

## 1 Introduction

Battery‐free wireless sensors operating in aquatic environments harness ambient energy sources—such as radio frequency (RF) fields, hydrokinetic energy, or acoustic energy—to continuously monitor environmental parameters in microfluidic devices, industrial pipelines, and natural water systems, providing real‐time data collection while eliminating the need for battery replacement and reducing maintenance requirements.^[^ ^1^, ^2^, ^3^, ^4^, ^5^, ^6^, ^7^, ^8^ ^]^ Near‐field communication (NFC) in passive mode facilitates simultaneous wireless data transmission and power delivery through a single link, simplifying system design, reducing connection requirements, and enabling compact integration into small and portable devices. The integration of NFC transponder with low‐power or passive sensors and near‐field antennas provides a sustainable and efficient solution for applications such as wearable electronics,^[^ ^9^, ^10^ ^]^ medical implants,^[^ ^11^, ^12^ ^]^ food quality monitoring,^[^ ^13^, ^14^ ^]^ and environmental monitoring.^[^ ^15^, ^16^ ^]^ While NFC technology is increasingly used in commercial applications like personal identification and content access, it has been seldom explored for underwater environments due to signal attenuation from water absorption and the impact of water on antenna spectral stability.^[^ ^17^, ^18^ ^]^ Operating at 13.56 MHz, the NFC system allows compact antenna designs, ideal for size‐constrained applications, while its near‐field properties provide enough power density for battery‐free operation, unlike radiative systems like Bluetooth and Wi‐Fi.^[^ ^19^, ^20^ ^]^ Even though NFC typically offers a short communication range of a few centimeters for data security, this short‐range capability allows effective contactless data transmission in challenging environments like conductive water. The wireless sensing platform in conductive aquatic environments, even at distances of several centimeters, shows great promise for a wide range of applications, including continuous environmental monitoring in industrial underground or subsea wellbore pipelines,^[^ ^21^ ^]^ as well as real‐time health monitoring and point‐of‐care testing.^[^ ^22^ ^]^

To ensure stable, secure, and uninterrupted sensor data transfer, efficient wireless power transfer is crucial for enabling battery‐free sensor nodes to perform sensing functions and communicate with the reader circuit. In an NFC system that relies on a non‐radiative evanescent magnetic field, power transfer depends on the inductive or magnetic resonant coupling between the reader and sensor antennas. Power transfer efficiency is primarily influenced by coupling strength, impedance matching, and the quality factors of the coupled antennas.^[^ ^23^, ^24^, ^25^, ^26^, ^27^ ^]^ These factors are affected by various elements, including antenna size and configuration, separation distance between antennas, frequency matching, operating frequency, and relative orientation of the antennas. Extensive methods have been proposed to enhance near‐field wireless power transfer, such as inserting a metamaterial slab or relay coils between the transmitter and receiver antennas to amplify magnetic flux density,^[^ ^28^, ^29^ ^]^ employing adaptive impedance‐matching networks to reduce power reflection,^[^ ^30^ ^]^ and utilizing various types of antennas—such as helical and spiral wire‐wound resonators,^[^ ^31^ ^]^ coils with a ferrite core,^[^ ^32^ ^]^ dielectric resonators,^[^ ^33^ ^]^ and cavity mode resonators^[^ ^34^ ^]^—with high Q‐factors to minimize ohmic and dielectric losses. Regarding wireless power transfer underwater, power loss arises not only from skin effects in the resonator and induced eddy currents in the conductive medium near the antenna but also from losses due to the penetration of the electric field into the surrounding medium. Besides power dissipation, the interaction between the antenna and the surrounding medium degrades the antenna's quality factor and shifts its resonance frequency. To mitigate power loss in the water while maintaining a high‐quality factor of the antenna, early efforts involved encapsulating the resonator in a high‐resistivity material to isolate the antenna from the conductive medium.^[^ ^35^, ^36^ ^]^ However, this approach increases the antenna's bulkiness, as the encapsulation layer must be sufficiently thick to ensure effective isolation. An alternative method involves inserting a solid ground plane between the antenna and the conductive medium to provide a short to ground. This configuration aims to terminate the electric field of the antenna before it penetrates the conductive medium, thereby eliminating losses due to electric field penetration.^[^ ^37^, ^38^ ^]^ However, this method has a drawback: the solid ground shield can induce an image current flowing in the opposite direction to that of the antenna, resulting in negative mutual coupling. This reduces the magnetic field and consequently decreases power transfer efficiency. A more advanced shielding method involves patterning the ground shield with slots perpendicular to the resonator traces. This patterned ground shield minimizes the impedance to the ground, providing effective termination for the electric field while increasing resistance to the image current and preventing negative mutual coupling.^[^ ^39^, ^40^ ^]^ Despite its effectiveness in reducing parasitic effects when used on human skin or silicon substrates, this approach is challenging when the antenna is immersed in a conductive medium. The patterned ground shield, which demonstrates spectral stability on only one side, does not fully address the issues of power transfer efficiency and quality factor degradation when submerged in conductive water.

Coaxial cables, with an inner conductor, a surrounding shield, and a dielectric layer in between, are widely used in RF applications due to their efficient signal transmission, low attenuation, and resilience against interference. The electromagnetic field is confined to the gap between the conductors, ensuring reliable performance. By strategically adding cuts to the inner and outer conductors and welding them at specific positions, coaxial cables can be engineered into electrically‐shielded resonators, effectively confining electric fields within the internal structural capacitance.^[^ ^41^, ^42^ ^]^ This design enhances spectral stability by shielding against background noise and parasitic coupling from nearby objects. Compared to patterned ground shield approaches, coaxially shielded resonators offer comprehensive omnidirectional protection with simple configurations, making them adaptable to diverse environments. This advantage has led to their application in conformable devices for biomedical imaging,^[^ ^43^, ^44^, ^45^, ^46^ ^]^ and wearable electronics.^[^ ^47^ ^]^ Previous studies have explored using these resonators for mid‐range wireless power transfer through magnetic resonance coupling, leveraging their predominant magnetic response and suppressed electric dipole moment. However, earlier efforts on coaxially shielded resonators for wireless power transfer have primarily focused on theoretical analysis and preliminary demonstrations with two resonators coupling in air. To the best of our knowledge, no existing system utilizes coaxially shielded resonators to fully harness their potential in conductive saltwater environments.

In this work, we developed a battery‐free system for underwater condition monitoring, enabled by coaxially shielded coils integrated into NFC sensor nodes. We first examined the configurations of two coaxially shielded coil resonators, analyzing their current profiles and electric field distributions in resonant states. This investigation demonstrated their spectral stability—particularly in resonance frequency and quality factor—when operating in aquatic environments with varying salinity levels. Using these electrically‐shielded coils, we developed a reader antenna enhanced by magnetic metamaterials and investigated its operating principles based on the coupled mode theory. We then experimentally mapped the magnetic field distribution in underwater environments, utilizing a small probe mounted on programmable motorized stages. Additionally, we designed a 3‐axis sensor antenna to alleviate the strict alignment requirements between the reader and sensor antennas.^[^ ^48^, ^49^ ^]^ By integrating this 3‐axis antenna with physical sensors and an NFC transponder, we created battery‐free wireless NFC sensor nodes capable of monitoring ambient light intensity and temperature in underwater conditions. Finally, we demonstrated the system's wireless sensing functionality, achieving a wireless communication distance of 54 mm in water with 3.5% salinity.

## 2 Results

### 2.1 System Overview

**Figure 1:**
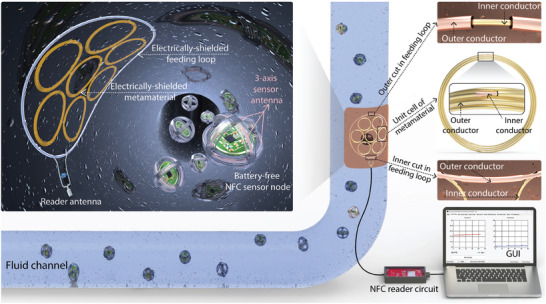
Illustration of the battery‐free wireless sensing system for underwater environmental monitoring.

**Figure** **1** illustrates the schematic of the battery‐free wireless sensing system designed for environmental monitoring within fluid pipeline channels. The system features a reader antenna composed of a single‐turn, coaxially shielded feeding loop with two cuts, paired with a magnetic metamaterial consisting of a hexagonally arranged array of multi‐turn, coaxially shielded resonators with a single cut. These resonators are mounted on an ultra‐thin, flexible polyimide substrate, which facilitates easy attachment to the curved pipeline wall. The large feeding loop and the uniformly distributed resonator array are engineered to redistribute the magnetic field in the region of interest, thereby expanding the antenna's reading area. The reader antenna connects to a commercial reader circuit via a coaxial cable, which is interfaced with a computer to display data collected from the NFC sensor nodes. On the sensor side, the NFC sensor nodes integrate an NFC transponder, a 3‐axis sensor antenna, and physical sensors for measuring light intensity and temperature within the pipeline fluid. The 3‐axis antenna comprises three orthogonally arranged, coaxially shielded resonators with a single cut. When fed by an external oscillator circuit, the reader antenna transmits RF power at 13.56 MHz to wirelessly power and interrogate the NFC sensor nodes as they move through the fluid channel. The sensor nodes harvest energy via magnetic resonance coupling as they pass through the reader antenna's coverage area. Once the energy transfer efficiency between the antennas reaches the threshold, the harvested voltage powers the NFC transponder and the sensor circuits, eliminating the need for batteries. The concept described has been experimentally implemented and is discussed in detail below.

### 2.2 Electrically‐Shielded Coils Crafted from Coaxial Cables

**Figure 2:**
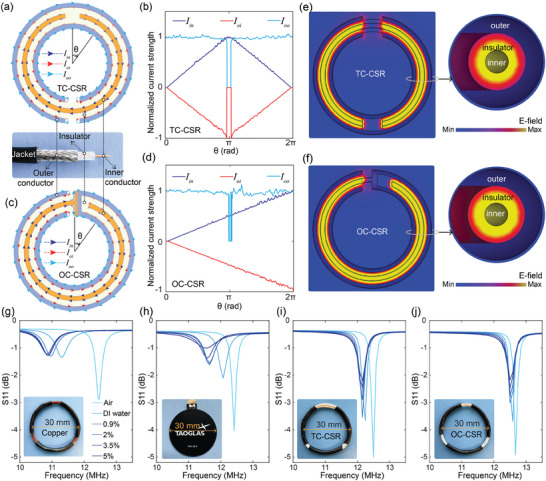
Electrically‐shielded coils. a) Configuration of a TC‐CSR and its electric current profiles along inner and outer conductors’ surfaces at resonance mode. b) Simulated electric currents’ magnitudes of the TC‐CSR. c) Configuration of an OC‐CSR and its resonating electric current profiles. d) Simulated electric currents’ magnitudes of the OC‐CSR. e,f) Simulated electric field patterns on the cutting plane of the TC‐CSR (e) and OC‐CSR (f). g–j) The variations of the reflection spectra of a homemade coil resonator made of copper wire (g), a commercial spiral resonator loaded with a capacitor (h), a TC‐CSR (i), and an OC‐CSR (j).

Before exploring the design of these antennas, we first examine the operation of electrically‐shielded coil resonators crafted from coaxial cables—the foundational components of the near‐field antennas—and their advantages over conventional resonators. To engage both the inner and outer conductors in resonance and create an electric shielding effect, cuts and welding points are strategically introduced in the conductors. **Figure** **2**a shows the configuration of the two‐cut coaxially shielded resonator (TC‐CSR), which is constructed from a loop of coaxial cable. In this configuration, a cut is introduced in the inner conductor, and a corresponding cut is made in the outer conductor at the opposite position. This configuration allows both conductors involved in the resonance to oscillate simultaneously. To analyze the electric field shielding properties of the TC‐CSR, the induced oscillating currents under external excitation can be categorized into three distinct components: the current along the inner conductor's surface *I_in_*, the current along the inner surface of the outer conductor *I_oi_*, and the current along the outer surface of the outer conductor *I_oo_*. For the TC‐CSR, the inner conductor is open‐ended due to the cut, causing *I_in_* to be zero at both ends and reach its maximum at the center. Strong coupling between the inner conductor and the inner surface of the outer conductor induces *I_oi_*, which flows in the opposite direction but with equal magnitude to *I_in_*. According to Kirchhoff's current law, at the cut in the outer conductor, *I_oi_* transitions from the inner to the outer surface of the outer conductor, forming *I_oo_*. The outer conductor's outer surface acts as a load, driven by the inner surface, and the current *I_oo_* maintains a constant magnitude equal to *I_oi_* at the cut area (refer to Figure S1, Supporting Information). Figure 2b presents the simulated average magnitudes of these currents using 3D electromagnetic simulation in CST Studio Suite, providing a quantitative comparison. Due to the opposing directions of *I_in_* and *I_oi_*, their respective magnetic fields cancel each other out, leaving *I_oo_* as the primary source of the magnetic field surrounding the TC‐CSR.

An alternative design of the electrically‐shielded coil, the one‐cut coaxially shielded resonator (OC‐CSR), involves cutting both the inner and outer conductors at the same location and welding one end of the inner conductor to one end of the outer conductor, as illustrated in Figure 2c. In the OC‐CSR configuration, the resonating current along the inner conductor increases linearly from zero at the open end to a maximum at the welded junction. Similar to the TC‐CSR, the induced current *I_oi_* flows in the opposite direction to *I_in_*, and at the outer conductor's cut, *I_oi_* flows to the outer surface, forming *I_oo_*, completing a closed current loop with *I_in_* at the welded point (refer to Figure S2, Supporting Information). Figure 2d displays the simulated surface current magnitudes across the conductors, offering a quantitative perspective. Same as the TC‐CSR, the magnetic fields generated by *I_in_* and *I_oi_* cancel out, leaving *I_oo_* as the sole contributor to the magnetic field near the resonator. For both TC‐CSR and OC‐CSR, the strategically positioned cuts in the inner and outer conductors generate substantial structural capacitance, enabling resonance despite the resonators’ small size relative to the RF wavelength in an NFC system. The key difference between the two designs lies in their effective inductance: the TC‐CSR's inductance is half that of the OC‐CSR, as indicated by the current profiles of *I_in_* in Figure 2b,d). As a result, while the resonance frequency for both resonators is determined by the coaxial cable segment length, the TC‐CSR resonates at twice the frequency of the OC‐CSR when both have the same length (refer to Figure S3, Supporting Information). The TC‐CSR's advantage is its two open ends, which can be connected to a feeding cable or circuit, whereas the OC‐CSR offers a more compact design due to its higher resonance frequency, making it a great candidate to fabricate metamaterials or resonator arrays working standalone, without the need to connect to outside cables.

The substantial inner structural capacitance generated by the coaxially aligned dual conductors within the resonators effectively confines the electric field within the resonator's inner structure. Figure 2e,f illustrate the electric field patterns on the axial cutting planes of the TC‐CSR and OC‐CSR under resonance conditions. Compared to the electric field in the gap between the inner and outer conductors, the electric field in the vicinity of the resonators is negligible. This unique property of the TC‐CSR and OC‐CSR arises from the strategically introduced cuts and welding points on the inner and outer conductors. In contrast, a closed ring resonator without cuts or a split ring resonator with cuts at the same locations on the inner and outer conductors does not exhibit electric field confinement in the gap between the conductors (refer to Figures S4 and S5, Supporting Information). This confinement is crucial for applications in aquatic environments, resulting in substantial attenuation of the electric field near the resonators and reducing power dissipation caused by electric field penetration into conductive water. Additionally, this attenuation diminishes parasitic capacitance between the resonator and the surrounding water, enhancing spectral robustness by stabilizing the resonance frequency and maintaining a high‐quality factor. To experimentally validate these findings, we conducted a comparative experiment using a homemade resonator crafted from copper wires, a commercial spiral resonator loaded with a capacitor, a TC‐CSR, and an OC‐CSR. The fabrication processes for the TC‐CSR and OC‐CSR are illustrated in Figures S6 and S7 (Supporting Information). All resonators had the same diameter and were tuned to the same resonance frequency, each sealed with a thin film to prevent direct contact with water (see Figure S8, Supporting Information). We immersed these resonators in water with varying salinity levels and measured their reflection coefficients, with results plotted in Figure 2g–j. For the TC‐CSR, the resonance frequency dropped from 12.5 to 12.25 MHz when transferred from air to deionized (DI) water, further decreasing by 0.09 MHz as salinity increased from 0% to 5%. The OC‐CSR exhibited a resonance frequency decrease of 0.08 MHz upon transitioning from air to DI water, followed by an additional reduction of 0.09 MHz with increasing salinity. In contrast, the traditional copper wire resonator showed a frequency detuning of 1.12 MHz when transitioning from air to water and a further decrease of 0.53 MHz with increasing salinity. Similarly, the commercial spiral resonator exhibited frequency reductions of 0.34 and 0.51 MHz, respectively. A smaller frequency variation due to changes in the environmental medium indicates greater stability of the resonance frequency. While the resonating strength of all resonators diminished when transitioning from air to water and further decreased with increasing salinity, the electrically‐shielded resonators demonstrated higher quality factors, as evidenced by the sharper dip at their resonance frequency in the reflection spectra. The combination of a stable resonance frequency and a high‐quality factor ensures superior performance of these resonators in wireless power transfer systems. This robustness allows the resonator to maintain a strong oscillating state at resonance, enabling stronger interaction with RF fields and enhancing the efficiency of RF field transmission and reception.

### 2.3 Reader Antenna Design

**Figure 3:**
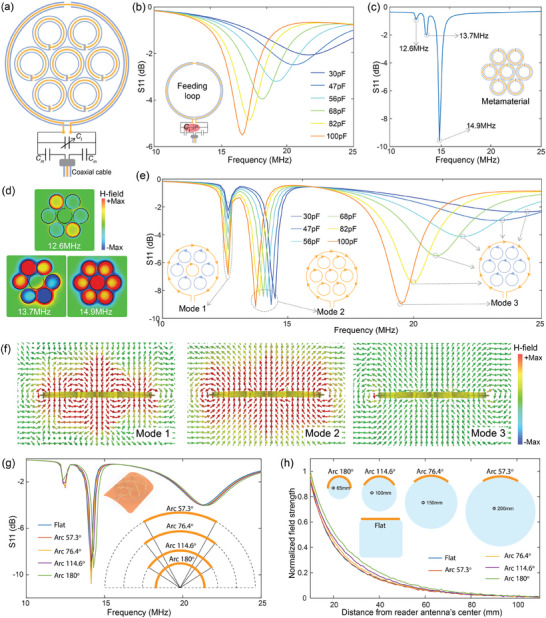
Electromagnetic characterizations of the reader antenna. a) Configuration of the reader antenna. b) Reflection spectra of the feeding loop as the tuning capacitance is varied. The inset shows the configuration of the feeding loop, constructed from TC‐CSR, along with a matching and tuning circuit. c) The reflection spectrum of the metamaterial within the reader antenna. The inset illustrates the configuration of the metamaterial, composed of an OC‐CSR array. d) Magnetic field patterns on the cutting plane of the metamaterial at different resonance frequencies. e) Reflection spectra of the reader antenna while sweeping the tuning capacitance. f) The magnitude and direction of the magnetic field across the various resonating modes of the reader antenna. g) Reflection spectra of the reader antenna with various curvatures. h) Magnetic field strength decay rate for the reader antenna with various curvatures.

Leveraging the robustness of the TC‐CSR and OC‐CSR against extraneous surrounding media, we propose a novel reader antenna by combining these two electrically‐shielded resonators. The reader antenna incorporates a single‐turn TC‐CSR, which has two open ends at the inner conductor's cut that allow connection to a tuning capacitor and matching circuit and is fed by an RF generating circuit through a coaxial cable. Inside the feeding loop, we integrate a metamaterial composed of an array of multi‐turn OC‐CSRs to redistribute the magnetic field in the vicinity of the reader antenna, effectively expanding the wireless reading coverage, as shown in **Figure** **3**a. To better visualize the antenna's internal configuration, we present a schematic drawing that simplifies the metamaterial's unit cell by omitting the number of turns. The properties of the reader antenna can be characterized by separately analyzing the spectral responses of the feeding loop and the metamaterial. First, for the feeding loop, we measured its reflection spectrum by connecting it directly to a vector network analyzer (VNA). By varying the tuning capacitor 𝐶_𝑡_, we could shift the resonance frequency from 21.59 to 16.63 MHz as 𝐶_𝑡_ increased from 30 to 100 pF as shown in Figure 3b, ensuring precise frequency matching with NFC technology. We then exited the metamaterial using an excitation loop connected to the VNA and measured its frequency response. The reflection spectrum is shown in Figure 3c. According to coupled mode theory,^[^ ^50^ ^]^ the interaction between OC‐CSRs in the metamaterial creates synergies that produce effective bulk properties, giving rise to multiple resonance states at different frequencies. As indicated by the dips in the reflection spectrum, three distinct resonance modes are excited by the excitation loop. Each mode corresponds to a different direction and strength of oscillating electric currents induced in individual OC‐CSRs, as illustrated by Figure 3d. In designing the reader antenna, identifying the resonance frequency at which the induced electric currents in each OC‐CSR align in the same direction and exhibit substantial oscillation magnitudes is essential for enhancing the RF field. Therefore, the highest resonance frequency is selected as the working mode of the metamaterial, where the current directions are aligned, and oscillation strength is maximized. After integrating the feeding loop with the metamaterial, the reader antenna was finalized. We measured the reflection spectra of the reader antenna under varying tuning capacitances, as depicted in Figure 3e. Due to the coupling between the feeding loop and the metamaterial, as well as the inner interactions between the metamaterial unit cells, three distinct resonance modes were observed for the reader antenna. The resonance current directions for these modes are illustrated in the Figure 3f. Unlike the other two modes, which exhibit opposite current directions, the second mode—dominated by the metamaterial's highest frequency resonance—features currents flowing in the same direction within both the unit cells of the metamaterial and the feeding loop. This alignment results in the superposition of the induced magnetic fields from each resonator, establishing this mode as the designated working mode of the reader antenna. Although the resonance frequency of the metamaterial itself is not tunable, the strong interaction between the feeding loop and the metamaterial can induce a shift in the metamaterial's resonance frequency. By fine‐tuning 𝐶𝑡, we can adjust the resonance frequency of the feeding loop, which in turn shifts the metamaterial's resonance frequency to the desired value. Consequently, leveraging the coupling between the feeding loop and the metamaterial, the overall working mode frequency of the reader antenna can be tuned from 14.45 to 13.638 MHz. Furthermore, using the ‐3 dB bandwidth method, which indicates that a narrower bandwidth corresponds to a more selective and higher‐quality resonator, the working mode demonstrates a much higher quality factor compared to the other two modes, as indicated by the reflection spectra. Additionally, adjusting the tuning capacitor affects only the resonance frequency, with minimal impact on the quality factor of the working mode. To address sensing requirements for pipelines or containers with varying diameters, we investigated the impact of antenna curvature on its resonance frequency and magnetic field decay rate. The antenna's reflection spectra were measured at curvatures of 57.3°, 76.4°, 114.6°, 180°, and in a flat configuration, as shown in Figure 3g. Results indicate that shifting from a flat to a semi‐cylindrical shape caused only a minor 0.15 MHz change in resonance frequency, demonstrating the antenna's stability across curvatures. We also examined magnetic field strength decay as a function of distance from the reader antenna's center, as shown in Figure 3h. The findings reveal that curvature has a limited effect on magnetic field strength in the surrounding area. Notably, rolling the antenna into a more pronounced curvature slightly increased magnetic field strength, attributed to the superposition of magnetic fields from opposite sides of the coil within the metamaterial. These results highlight the antenna's consistent electromagnetic performance under varying curvatures, beneficial for flexible sensing in dynamic environments.

### 2.4 Magnetic Field Mapping in Underwater Environments

**Figure 4:**
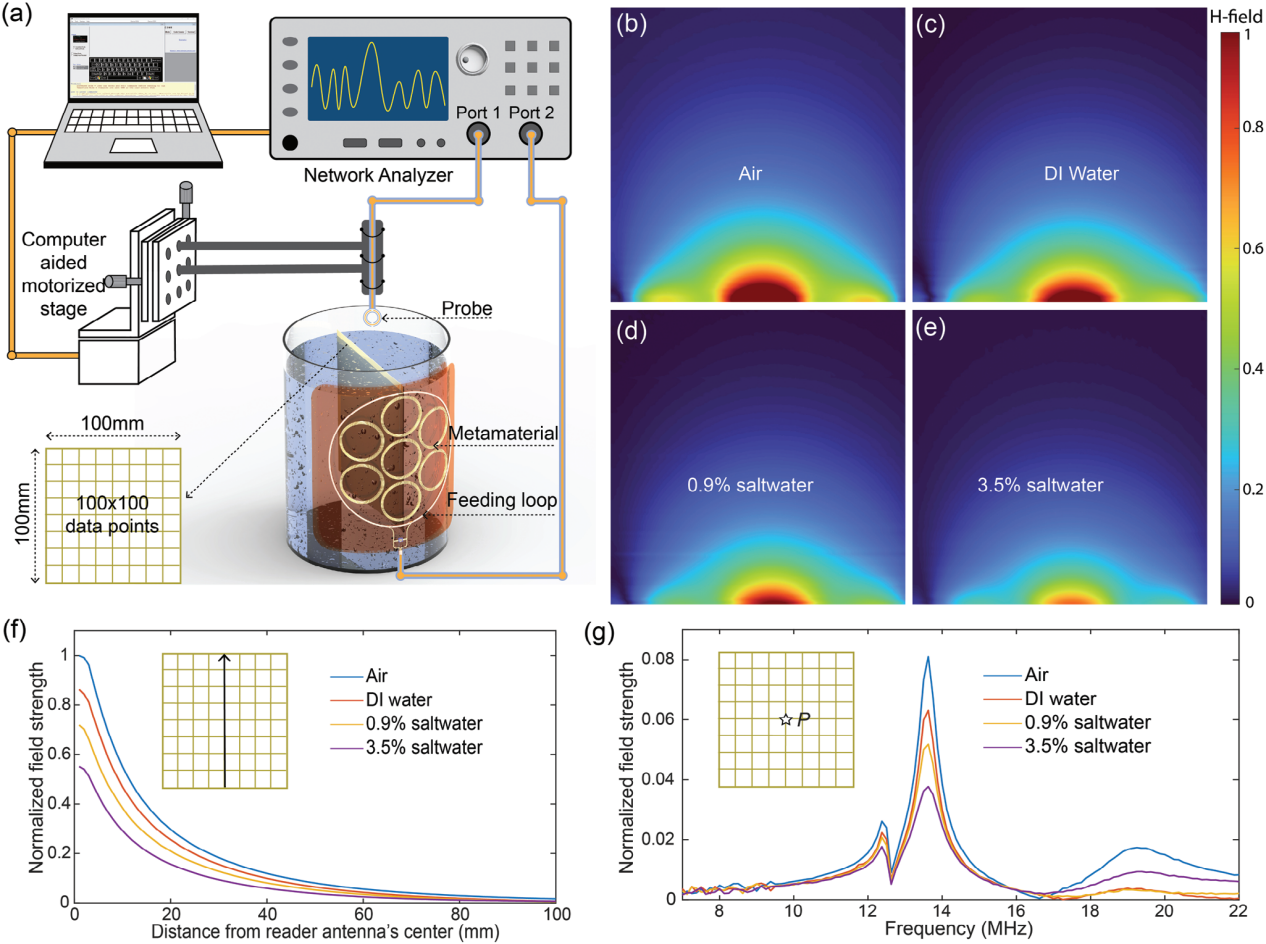
Magnetic field mapping in underwater environments. a) Illustration of the magnetic field mapping setup. b–e) Measured magnetic field strength on the cross‐section (indicated by the yellow grid in (a)) when the communication medium is air (b), DI water (c), 0.9% saltwater (d), and 3.5% saltwater (e). f) The decay rate of the magnetic field strength in the four communication medium as moving away from the reader antenna. g) Spectra of the magnetic field distribution at location (indicated by the pentagram in the inset figure) with separation distance from the reader antenna of 50 mm.

The underwater environment, particularly saltwater with its high conductivity, presents substantial challenges to RF field propagation. To determine the optimal coil configurations and shielding techniques for enhancing power transfer efficiency and stability, we systematically mapped the magnetic field distribution in both air and water with varying salinity using a near‐field mapping system, as illustrated in **Figure** **4**a. In this setup, the reader antenna was wrapped around the surface of the water container and connected to port 1 of the VNA, with the RF signal fed into the feeding loop serving as the excitation wave. A small TC‐CSR probe was connected to port 2 of the VNA via a coaxial cable and mounted on a computer‐controlled motorized 2D stage, allowing precise data collection in the underwater environments. Measurements were taken across 100 × 100 points over a 100 × 100 mm area, with the transmission coefficient (S21) used to measure magnetic field strength on the cutting plane, represented by the yellow grid in the water container. The normalized magnetic field patterns on the yellow plane for different mediums—air, and water with salinity levels of 0%, 0.9%, and 3.5%—are shown in Figure 4b–e. Compared to the magnetic field pattern in air, water environments cause attenuation of the field strength due to the interaction between the RF field and the surrounding medium. This attenuation becomes more pronounced as the salinity level increases, with both conductive and dielectric losses intensifying, leading to greater power dissipation. We also extracted the magnetic field strength along the central line of the cutting plane to quantitatively assess variations in field strength as a function of distance from the surface of the reader antenna, as plotted in Figure 4f. The transition from air to water results in a decrease in magnetic field strength of 22%, 36%, and 53% for salinity levels of 0%, 0.9%, and 3.5%, respectively. Additionally, the spectra of the magnetic field strength at the location 50 mm from the reader antenna (point *P* on the cutting plane) are shown in Figure 4g. The magnetic field reaches its peak at the resonance frequency across all salinity conditions, with any deviation from this frequency substantially reducing field strength. For example, a 0.5 MHz shift from resonance results in a field strength decrease of 61.5%, 60.5%, 55.8%, and 47% for air, 0% salinity, 0.9% salinity, and 3.5% salinity, respectively. These quantitative results reveal that a 0.5 MHz frequency shift dramatically impacts the field strength, directly leading to a decrease in power transfer efficiency. To ensure high power transfer efficiency, it is essential to maintain the antenna's resonance frequency at the designated 13.56 MHz, as used in this work based on NFC technology. In addition, the discrepancy in the field strength decrease due to the 0.5 MHz shift indicates that water environments, particularly those with high salinity, extend the bandwidth, which corresponds to a reduction in the antenna's quality factor. These findings have enabled us to analyze the effects of salinity on RF field distribution and develop strategies to mitigate attenuation, thereby ensuring reliable wireless power transfer for battery‐free systems in diverse aquatic environments.

### 2.5 Sensor Antenna Design and Characterization

**Figure 5:**
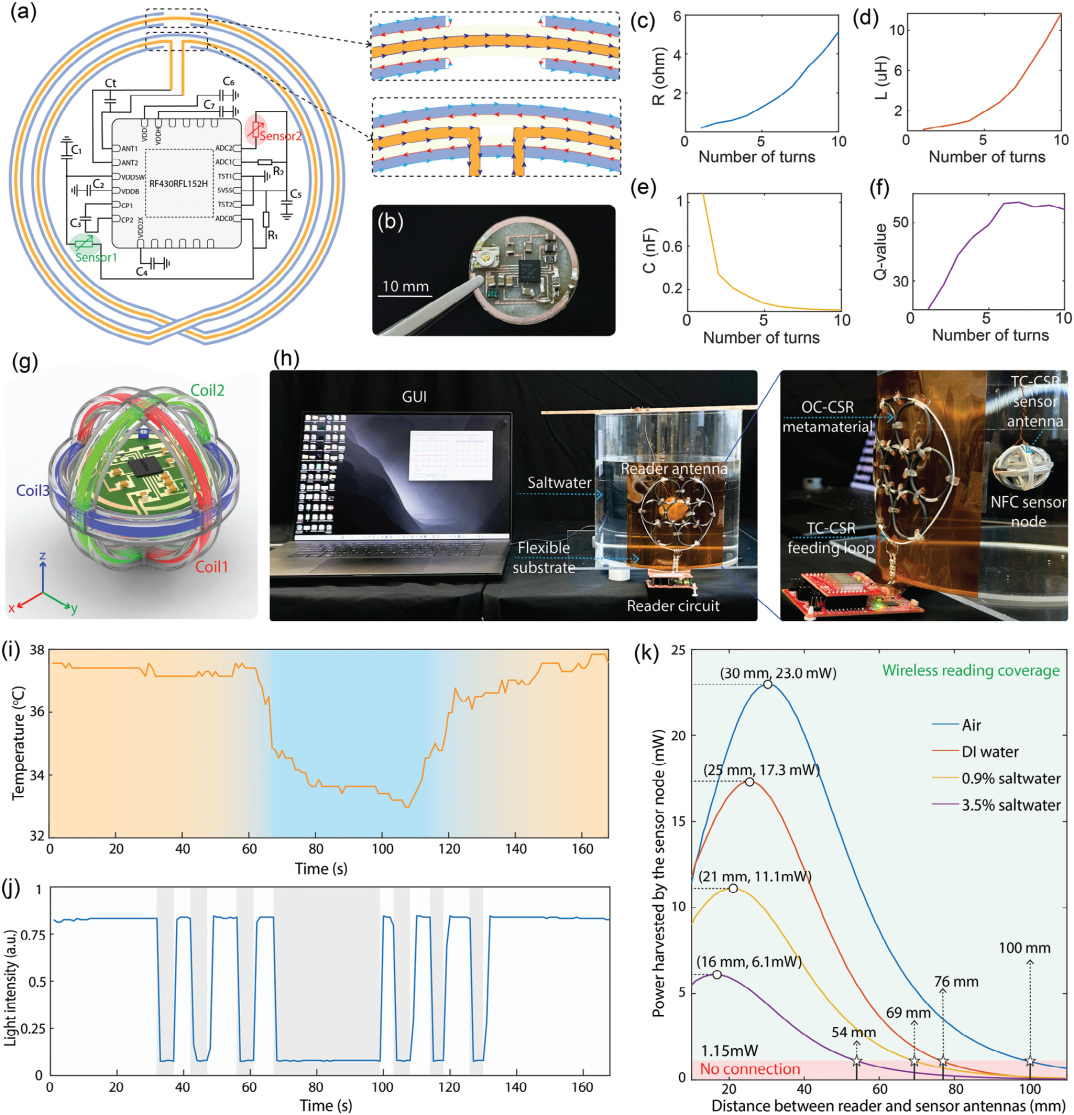
NFC sensor node design and experimental validation. a) The configuration of the NFC sensor circuit is integrated with a TC‐CSR‐enabled sensor antenna. b) The photo of the NFC sensor circuit. c–f) Variations of the effective resistance (c), inductance (d), capacitance (e), and quality factor (f) of the sensor antenna made by a TC‐CSR loaded with a capacitor as sweeping the length of the coaxial cable of the TC‐CSR. g) Illustration of NFC sensor node integrating with 3‐axis sensor antenna. h) The experimental setup for the battery‐free wireless sensing for underwater environmental monitoring. i) The measured water temperature as pouring hot water and ice into the container. j) The measured ambient light intensity by switching on and off of the light. k) Quantitative analysis for the power harvested by the sensor node.

In this work, the wireless sensor nodes are developed based on the NFC transponder RF430FRL152H. A key feature of this transponder is its passive communication and sensor measurement capabilities with low power consumption, allowing the NFC sensor nodes to operate directly from energy harvested by the sensor antennas. Additionally, the transponder integrates three analog‐to‐digital converters (ADCs), enabling simultaneous measurement of multiple parameters through different sensors, such as the temperature and light sensors employed in this study. The high level of integration makes the sensor node compact, multifunctional, and low‐profile. A critical component of the sensor node is the antenna, which plays a vital role in both energy harvesting and wireless communication between the sensor node and the reader circuit. For improved spectral stability in conductive environments, the TC‐CSR is employed as the sensor antenna. Its two open ends at the inner conductor's cut provide a direct RF feed to the NFC transponder circuit. **Figure** **5** **a** illustrates the connection between a TC‐CSR and the NFC circuit, while Figure 5b shows a photo of the fabricated NFC circuit (see Table S1, Supporting Information for the bill of materials for the NFC sensor circuit). To maximize the performance of the TC‐CSR, we first optimized its size and configuration. Considering the dimensions of the NFC circuit and the fluid channel, the TC‐CSR diameter was set to 25 mm with multiple turns, as more turns reduce the resonance frequency by increasing the inductance and capacitance of the coaxial cable. Based on our measurements, a TC‐CSR with approximately ten turns resonates ≈13.56 MHz. However, increasing the number of turns raises the internal resistance, potentially causing power dissipation and reducing the antenna's Q factor. Conversely, a shorter length decreases inductance, weakening the magnetic dipole and response. To identify the optimal length of the coaxial cable, we fabricated TC‐CSRs with varying numbers of turns, ranging from 1 to 10. To maintain resonance at 13.56 MHz, a tuning capacitor was added to the open ends of the inner conductor to compensate for the necessary capacitance. Using an impedance analyzer, we measured the effective resistance, inductance, capacitance, and Q‐value of each TC‐CSR configuration. The results, shown in Figures 5c–f, indicate that the optimal *Q*‐value occurs with 6 to 7 turns, balancing inductance and resistance. Thus, the finalized design features a 25 mm TC‐CSR with 7 turns as the sensor antenna. For wireless power transfer through magnetic resonant coupling, precise alignment between the reader and sensor antennas is essential for efficient power transfer. Angular deviations between the antennas can dramatically reduce transmission efficiency. In underwater environments, there is a potential for misalignment, as sensor nodes may not remain parallel to the reader antenna or may rotate as they pass through. To address this, we developed a 3‐axis sensor antenna, shown in Figure 5g, composed of three mutually orthogonal TC‐CSRs, each connected to the NFC transponder circuit. This 3‐axis configuration enhances continuous coupling with the reader and ensures stable energy harvesting from the magnetic field, even when the NFC sensor node rotates relative to the reader antenna, thus providing a more reliable power supply for the sensor circuit.

### 2.6 Proof‐of‐Concept Underwater Environmental Monitoring

To experimentally validate the potential of the electrically‐shielded coil in a battery‐free wireless sensing system for underwater environmental monitoring, we designed a proof‐of‐concept experimental setup, as illustrated in Figure 5h. In this setup, the reader antenna was attached to the outer surface of a cylindrical container with a diameter of 200 mm and a wall thickness of 3.5 mm. The metamaterial‐enabled antenna was powered by a reader circuit. The circuit was controlled by custom software through a graphical user interface (GUI), enabling full interaction with the sensor nodes. As the sensor nodes passed through or came within the reader antenna's coverage area, the reader circuit continuously interrogated them, and the GUI displayed real‐time measurements of temperature and light intensity within the container's medium. In the experiment, the sensor node was suspended inside the container, maintaining a 50 mm distance from the reader antenna. To fully explore the capabilities of the electrically‐shielded coils, we conducted tests in various media. We first tested in air within the empty container, followed by filling the container with DI water, and finally, adding salt to achieve a salinity level up to 3.5%, simulating saltwater conditions. To alter the temperature readings, we began with ≈37.5 °C water, added ice to cool it, and then poured in hot water to adjust the temperature. The temperature manipulation process was recorded and plotted in Figure 5i. Additionally, we validated the sensor node's ability to monitor light intensity by turning the room lights on and off, and adjusting the light within the container. The corresponding light intensity measurements are shown in Figure 5j, clearly illustrating the system's response to changes in lighting conditions. The results demonstrate the effectiveness of electrically‐shielded antennas in conductive underwater environments. Additionally, to demonstrate the system's ability to perform battery‐free wireless sensing while the sensor node is in motion, the sensor node was mounted on a motorized stage and moved in the water at a speed of approximately 10 cm s^−1^. Under these conditions, the communication link was successfully established. The maximum motion speed or fluid flow rate should be below 1 m s^−1^, as the sensor node's fastest sampling time is 0.1 s per data acquisition, and the reading coverage is approximately 100 mm. To quantitatively analyze the threshold power required to activate the sensor node and the power transfer efficiency between the reader and sensor node, we used a spectrum analyzer to directly monitor the harvested power by the sensor antenna. To characterize the harvested power, we varied the separation distance between the reader and sensor antennas under different environments. As shown in Figure 5k, with a transmitting power of 100 mW from the reader antenna, the maximum wireless sensing distances are 100 mm in air, 76 mm in DI water, 69 mm in 0.9% saltwater, and 54 mm in 3.5% saltwater. At the maximum reading distance, the harvested power is approximately 1.15 mW, representing the threshold power required to operate the sensor node. The optimal distances for maximum power transfer efficiency are 30 mm for harvesting 23 mW in air, 25 mm for 23 mW in DI water, 21 mm for 23 mW in 0.9% saltwater, and 16 mm for 23 mW in 3.5% saltwater, respectively. Interestingly, the optimal distance was not the shortest distance between the reader and sensor antennas. This is attributed to over‐coupling, where excessively close proximity causes a shift in the resonance frequencies of the two antennas, reducing power transfer efficiency. In saltwater, the conductive medium weakens the interaction between antennas, mitigating the severity of over‐coupling compared to air or DI water.

## 3 Discussion

We introduced an innovative near‐field antenna design for battery‐free wireless sensing and real‐time monitoring in complex environments. These antennas, based on electrically‐shielded coils crafted from coaxial cables, use strategic cuts and welding points in the conductors to generate inner structural capacitance, enabling resonance at NFC frequencies. The internal capacitance confines the electric field within the resonator, minimizing interactions with surrounding materials, reducing power dissipation in conductive environments, and enhancing spectral stability, maintaining both resonance frequency and quality factor. For the first time, we comprehensively explore the potential of electrically‐shielded near‐field antennas fully immersed in saltwater with salinity levels up to 3.5%. Building on previous studies of coaxially shielded resonators, this work remarkably broadens their application scope and offers new insights into the design of robust metamaterials and antennas for extreme environment monitoring. Since the electric field shielding properties of coaxially shielded resonators stem primarily from their unique dual‐layered internal conductors, the near‐field antenna characteristics remain theoretically valid across various configurations and coaxial cable sizes. The frequency of the shielding coils is determined by the length between the cuts in the inner and outer conductors, offering great design flexibility. For example, longer coaxial cables result in lower resonance frequencies, while shorter cables yield higher frequencies. Furthermore, by introducing multiple cuts along the coaxial cables, larger resonators can be fabricated that maintain high‐frequency resonance. This design flexibility makes the antenna highly adaptable for a wide range of near‐field applications, such as battery‐free underwater vehicles, epidermal electronics, transcranial magnetic stimulation, and wearable magnetic resonance imaging.

Future efforts will focus on developing advanced encapsulation techniques and sensor coatings to ensure long‐term stability in underwater environments. These coatings will provide hermetic sealing, corrosion resistance, biofouling protection, and buoyancy control, thereby enhancing system durability for extended deployments. Additionally, as a proof of concept, we have integrated temperature and light sensors into NFC transponders. Future work may involve incorporating different commercial or custom‐built sensors, such as pressure, flow, gas, or biosensors, to expand real‐time monitoring from industrial to personal healthcare applications. The trend toward miniaturization in modern systems highlights the importance of antenna miniaturization. Addressing the miniaturization of the electrically‐shielded coils is another key consideration for future research. Currently, the shielded coils are crafted from micro coaxial cables with inner and outer conductor diameters of 0.15 and 0.49 mm, respectively. Further reductions in coaxial cable size may introduce fabrication challenges. One potential solution is adapting the coaxially shielded coil configuration into a planar strip line structure by introducing slits along the transmission line, creating multilayer electrically‐shielded resonators compatible with MEMS fabrication processes. These miniaturized antennas could enable broader applications by fitting into space‐constrained environments and facilitating less invasive procedures while maintaining efficient wireless powering and communication.

## 4 Experimental Section

### Resonators Employed for Underwater Characterizations

The TC‐CSR and OC‐CSR shown in Figures 2i,j were constructed using micro coaxial cable (9436, Alpha Wire), with conductor, insulation, and shield diameters measuring 0.15, 0.38, and 0.49 mm, respectively. The copper resonator in Figure 2g was fabricated from enameled magnet copper wire with the diameter of 0.32 mm. The commercial flexible NFC antenna with a reverse ferrite layer (Taoglas) in Figure 2h was loaded with a capacitor to ensure resonance ≈12.5 MHz. All resonators were designed to have the same resonance frequency and a uniform diameter of 30 mm to enable fair comparison.

### Reader and Sensor Antennas Fabrication

The reader antenna comprise a feeding loop paired with metamaterials at its center. The feeding loop was constructed from a TC‐CSR with a diameter of 100 mm and was equipped with a tuning and matching circuit that included a matching capacitor with a capacitance of 68 pF. Inside the feeding loop, the metamaterial consisted of seven hexagonally packed OC‐CSRs, each with a diameter of 30 mm, separated by a distance of 30 mm. The 3‐axis sensor antenna was loaded with a 33 pF capacitor to ensure that its resonance frequency was compatible with the NFC transponder.

### NFC Sensor Node Construction

The miniaturized NFC sensor circuit was fabricated on an RF‐4 substrate using a PCB milling machine (ProtoMat S64, LPKF), with a board diameter of 20 mm. This sensor node circuit integrated a commercial NFC transponder (RF430FRL152H, Texas Instruments), a thermistor (B57471V2104J062, TDK Electronics), and an ambient light sensor (TEMT6200FX01, Vishay Semiconductors). For light intensity measurements, a 430 kΩ resistor (R1) was chosen for the voltage divider circuit, while a 100 kΩ resistor (R2) was used for temperature measurement, and together with a properly programmed sampling process of the NFC transponder, they ensured the accuracy and resolution of the data acquired from both sensors. The circuit board, along with the 3‐axis sensor antenna, is housed within a 3D‐printed enclosure. The temperature sensor had a sensitivity of 0.27 °C, with a detection range of 15–50 °C. For the light sensor, the sensitivity was 78.1 lx, and the detection range spans from 10 to 10,000 lx. The sensor node's response time was programmable and set to 0.1 s, with an adjustable range from 0.1 to 6.2 s for each data acquisition. A faster response time reduced the bit accuracy of the internal ADCs. The sampling accuracy of NFC chip was programmable to be 7, 9, 10, 12, 13, 15, and 16‐bit, which corresponds to a sampling time of 32, 64, 128, 256, 1024, and 2048 ms, respectively.

### Electromagnetic Characterizations

All numerical simulations were performed using the frequency domain solver in CST Microwave Suite. The simulation models were scaled up from the actual size of the coaxial cables, utilizing larger diameters to enhance the visualization of surface current profiles and electric field patterns in the gaps between the inner and outer conductors. The reflection spectra, illustrated in Figures 2 and 3, were measured using a vector network analyzer (VNA, E5071C, Keysight Inc.) equipped with a circular excitation loop. For the magnetic field mapping presented in Figure 4, the probe was constructed using a non‐resonant TC‐CSR with a diameter of 5 mm. This size was carefully selected to ensure it was small enough to minimize its impact on the field distribution of the reader antenna, yet large enough to be effectively excited by the magnetic field in water, allowing for accurate measurements at distances further from the reader antenna. The Q‐factor, effective inductance, capacitance, and resistance, depicted in Figure 5, were measured using an impedance analyzer (IM7581, Hioki). The experiments to monitor the power harvested by the sensor antenna from the reader circuit were conducted using a spectrum analyzer (Spectrum Analyzer, N9322C, Keysight Inc), with the reader fixed in position and the sensor antenna connected to the spectrum analyzer. The sensor antenna was mounted on a computer‐controlled motorized stage, which precisely controlled the separation distance between the reader and sensor antennas. In all experiments involving the submersion of devices in water with varying salinity levels, the resonators, antennas, and circuits were sealed with a polyurethane film to prevent direct contact between the conductive solution and sensitive components.

### Data Readout of Underwater Environmental Monitoring

Sensor data readout was performed using an NFC reader circuit, consisting of an MSP‐EXP430G2 Launchpad, integrated with a TRF7970A Booster Pack. The Booster Pack was modified to enable the connection of the metamaterial‐enhanced reader antenna, which improves signal sensitivity and communication range. Data transmission between the reader circuit and the sensor nodes was facilitated using the ISO 15693 protocol. The reader circuit was powered via a USB cable connected to a computer, which ran a custom graphical user interface (GUI). This GUI provided user interaction with the sensor nodes, allowing for programming the sampling process, real‐time monitoring of sensor data, visual display of measurements, and options for data export and analysis.

## Conflict of Interest

The authors declare no competing interests.

## Supporting information



Supporting Information

## Data Availability

The data that support the findings of this study are available from the corresponding author upon reasonable request.
