# Supplementary material for: Electrically‐Shielded Coil‐Enabled Battery‐Free Wireless Sensing for Underwater Environmental Monitoring
Source: Adv Sci (Weinh). 2025 Jan 31;12(14):2414299. doi: 10.1002/advs.202414299 (PMC11984836; doi:10.1002/advs.202414299)
Supplement: Supplementary file 1 — Supporting Information [file ADVS-12-2414299-s001.pdf]

ADVANCED SCIENCE
Open Access

Supporting Information

for *Adv. Sci.*, DOI 10.1002/advs.202414299

Electrically-Shielded Coil-Enabled Battery-Free Wireless Sensing for Underwater Environmental Monitoring

*Ke Wu, Xia Zhu, Stephan W. Anderson and Xin Zhang**

Supporting Information

Electrically-Shielded Coil-Enabled Battery-Free Wireless Sensing for Underwater Environmental Monitoring

*Ke Wu, Xia Zhu, Stephan W. Anderson, and Xin Zhang**

K. Wu, X. Zhu, X. Zhang

Department of Mechanical Engineering, Boston University, Boston, MA 02215, United States.

E-mail: xinz@bu.edu

S. W. Anderson

Chobanian & Avedisian School of Medicine, Boston University Medical Campus, Boston, MA, 02118, United States.

K. Wu, X. Zhu, S. W. Anderson, X. Zhang

Photonics Center, Boston University, Boston, MA 02215, United States.

This file includes:

Figures: S1 to S8

Table S1

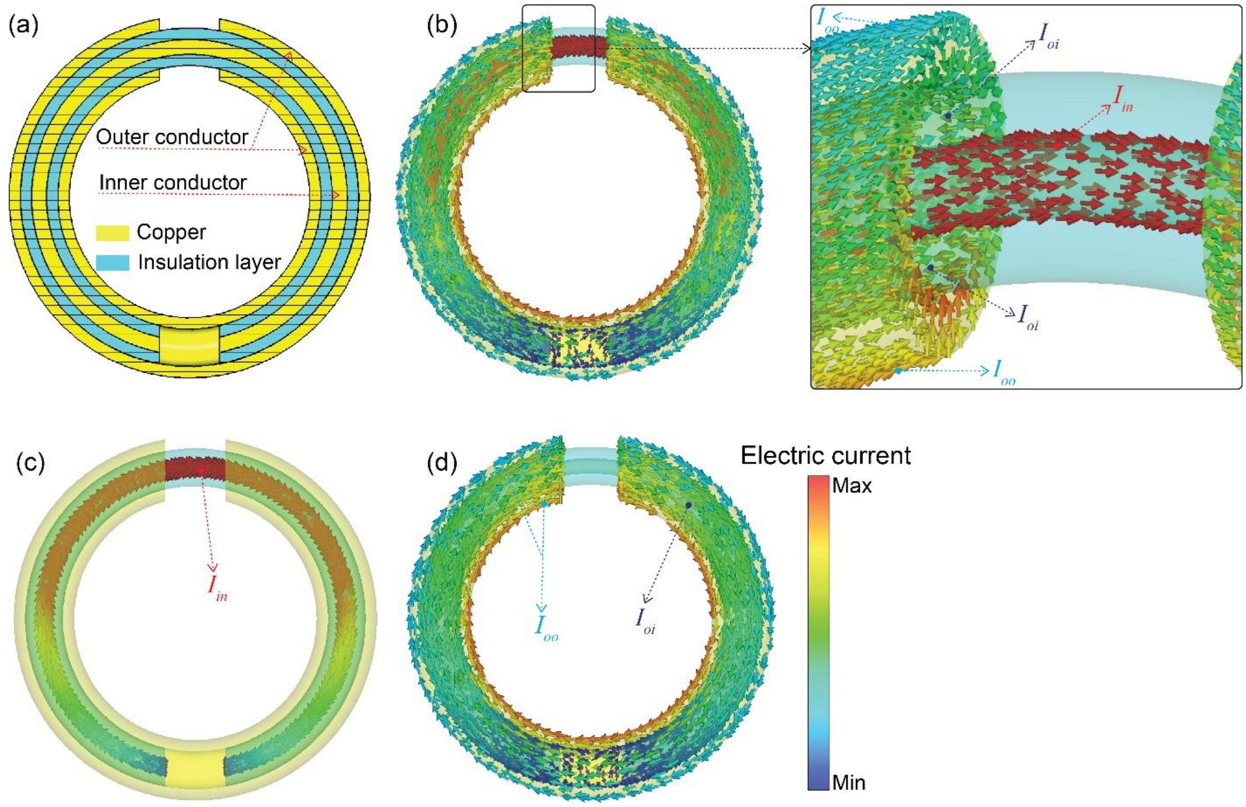

Figure S1. Simulated electric current profiles of the TC-CSR in the resonating state. (**a**) Cross-sectional view of the simulation model. (**b**) Simulated electric current distribution on the surfaces of the inner and outer conductors. (**c**) Revelation of electric current I_{in} with the outer conductor hidden. (**d**) Revelation of electric currents I_{oi} and I_{oo} with the inner conductor hidden.

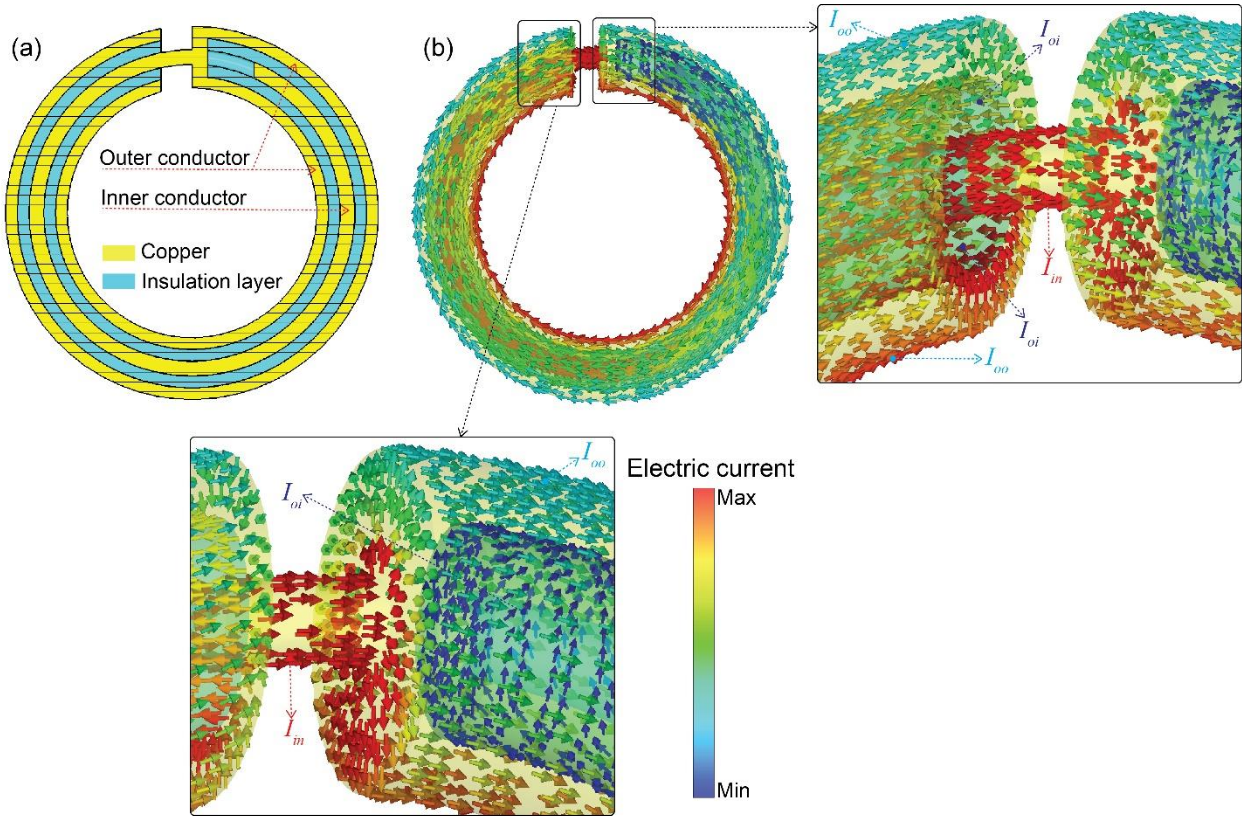

Figure S2. Simulated electric current profiles of the OC-CSR in the resonating state. (**a**) Cross-sectional view of the simulation model. (**b**) Simulated distribution of electric currents I_{in}, I_{oi}, and I_{oo} on the surfaces of the inner and outer conductors.

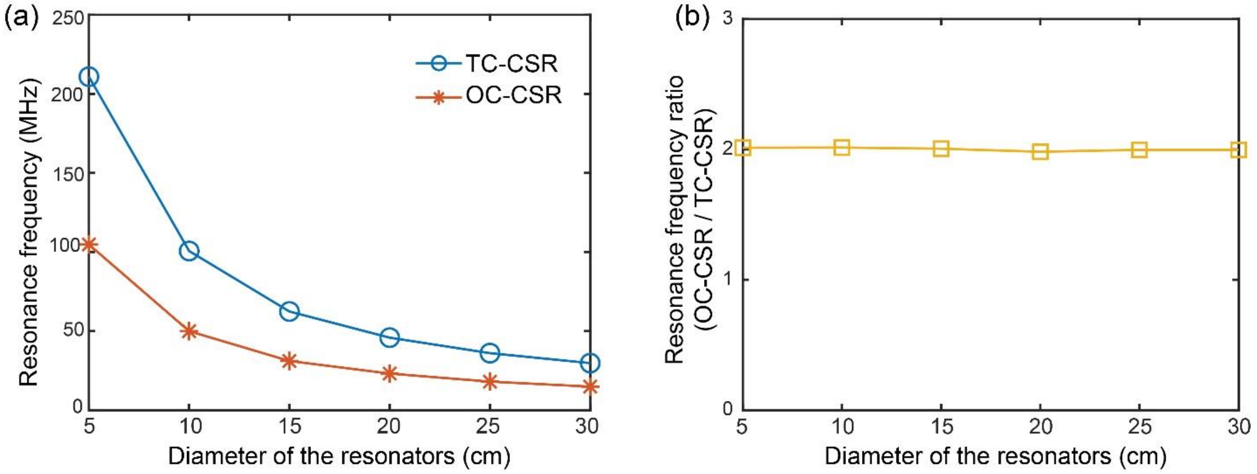

Figure S3. Correlation between the resonance frequency and the total cable length of the coaxially shielded resonators. (**a**) Resonance frequencies of the single-turn TC-CSR and OC-CSR as a function of the resonator diameter. (**b**) Resonance frequency ratio between OC-CSR and TC-CSR as the resonator diameter is varied.

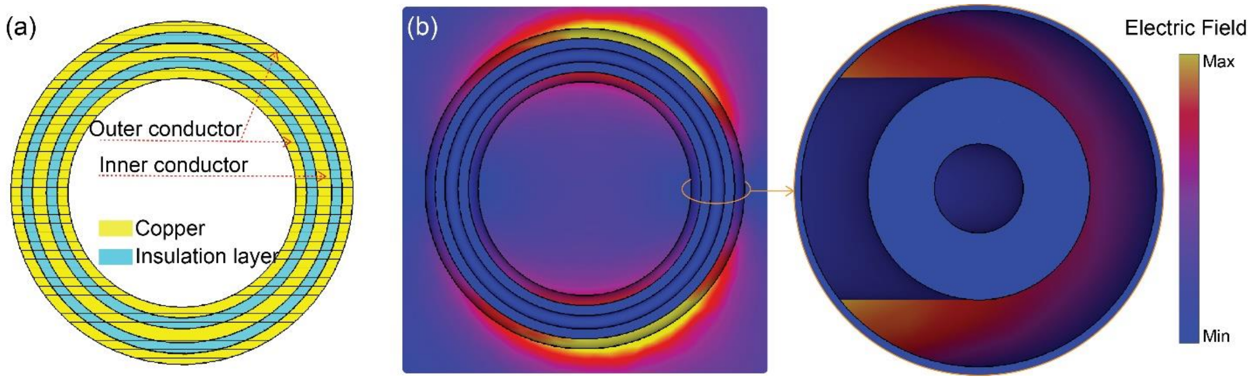

Figure S4. Simulations of the closed ring resonator made from a coaxial cable without cuts. (**a**) Cross-sectional view of the simulation model. (**b**) Simulated electric field distribution of the closed ring resonator.

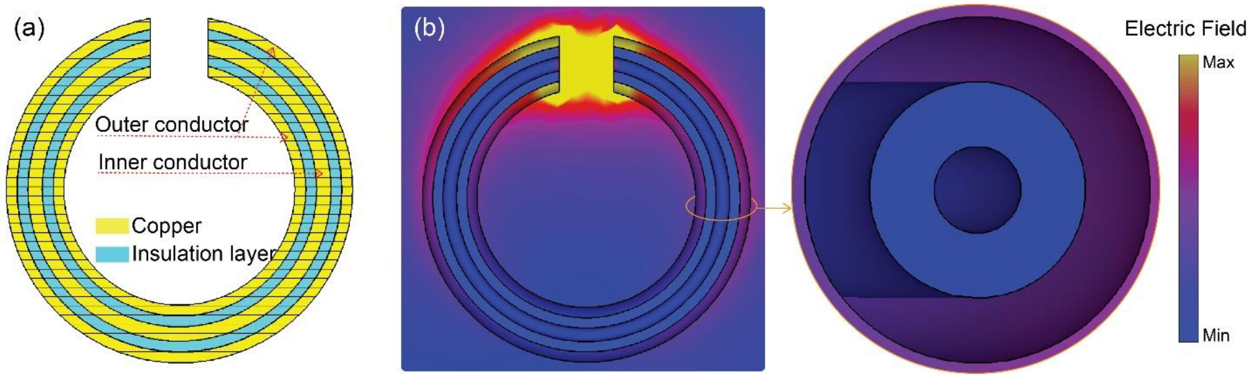

Figure S5. Simulations of the split ring resonator made from a coaxial cable. (**a**) Cross-sectional view of the simulation model. (**b**) Simulated electric field distribution of the split ring resonator.

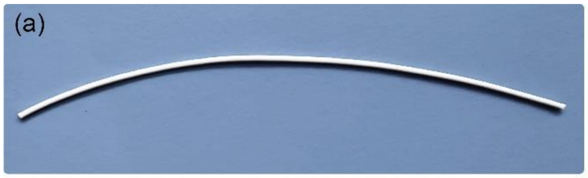

Prepare a sement of coaxial cable.

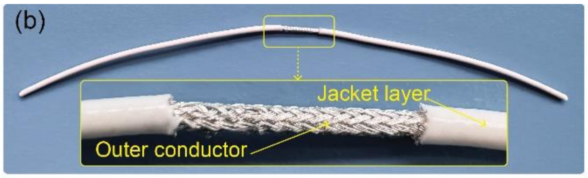

Peel off the jacket layer in the middle of the coaxial cable to expose the outer braid conductor.

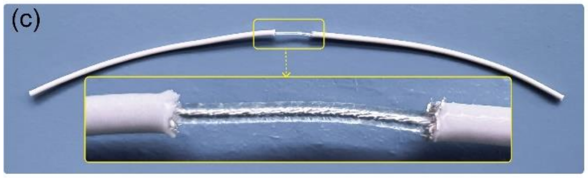

Cut away the exposed outer conductor to reveal the inner conductor, which is encased in a transparent insulation layer.

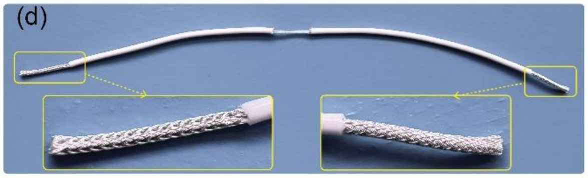

Peel off the jacket layer at both open ends of the coaxial cable.

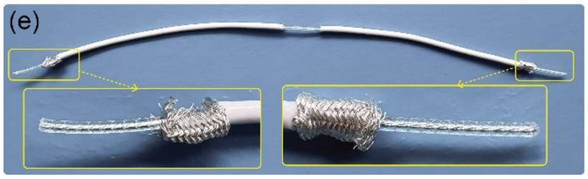

Roll up the exposed outer conductor to reveal the inner conductor at both open ends.

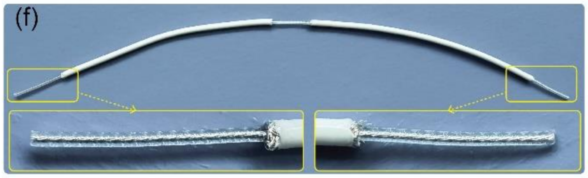

Cut away the outer conductor at both open ends.

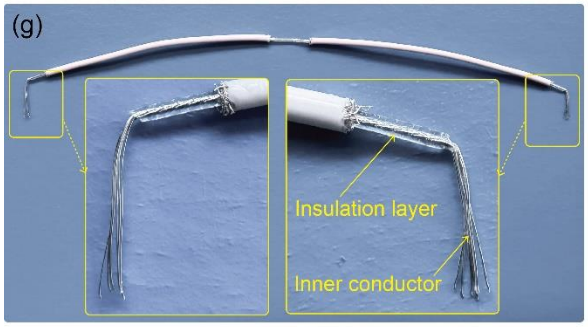

Remove the insulation layer surrounding the inner conductor at both open ends.

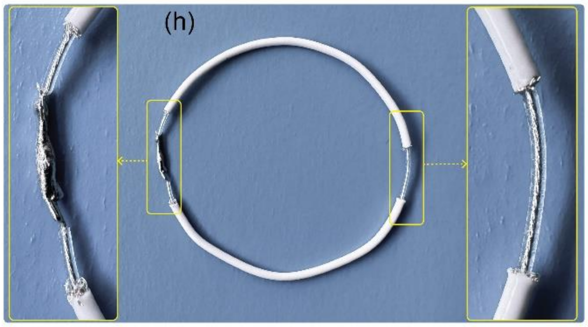

Weld the inner conductors together at both open ends to form the two-cut coaxially shielded resonator (TC-CSR).

Figure S6. Fabrication process of the two-cut coaxially shielded resonator (TC-CSR).

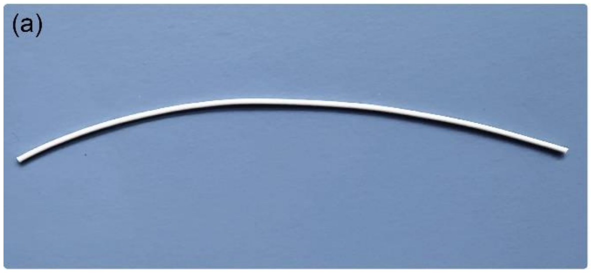

Prepare a sement of coaxial cable.

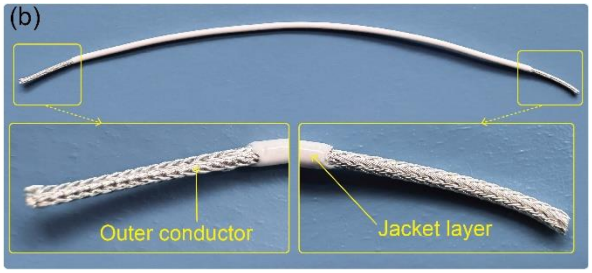

Remove the jacket layer from both two open ends of the coaxial cable to expose the outer conductor.

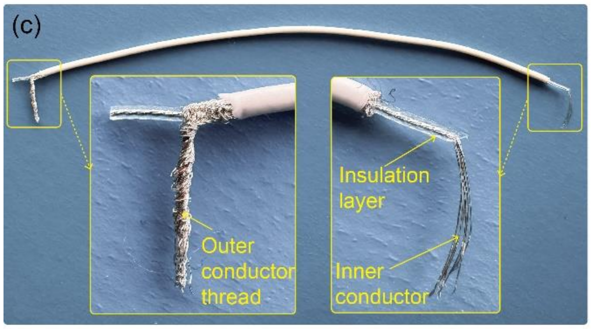

Twist the exposed outer conductor into a thread and trim the inner conductor at one end of the coaxial cable. At the other end, remove the outer conductor and strip away the insulation layer covering the inner conductor to fully expose it.

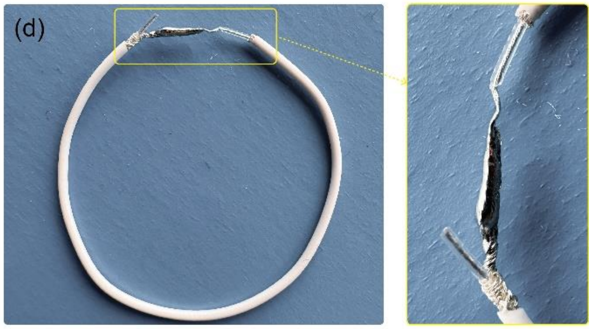

Weld together the inner conductors at one end and the outer conductor at the other end to form the one-cut coaxially shielded resonator (OC-CSR).

Figure S7. Fabrication process of the one-cut coaxially shielded resonator (TC-CSR).

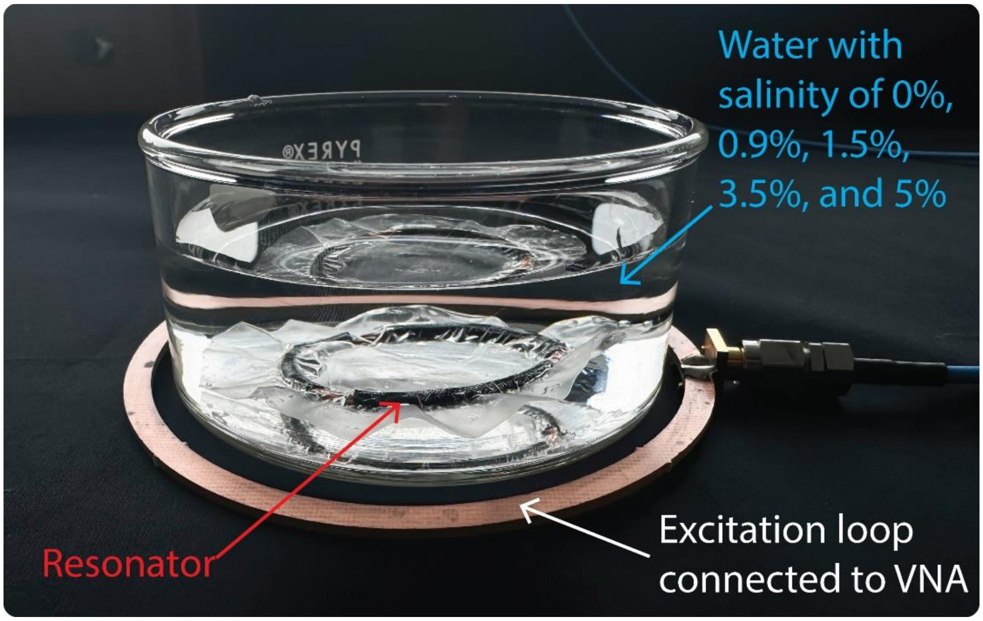

Figure S8. Experimental setup for characterizing the electromagnetic properties of resonators in underwater environments.

Table S1. Bill of materials of the NFC sensor node.

**Components**	**Description**	**Value**
Integrated chip	*NFC 15693 sensor transponder*	*RF430RF152H, Texas Instruments*
Sensor 1	*Thermistor*	*B57471V2104J062, TDK Electronics*
Sensor 2	*Ambient light sensor*	*TEMT6200FX01, Vishay Semiconductors*
C_1	*Capacitor*	*2.2μF*
C_2	*Capacitor*	*0.1μF*
C_3	*Capacitor*	*10nF*
C_4	*Capacitor*	*0.1μF*
C_5	*Capacitor*	*1μF*
C_6	*Capacitor*	*0.1μF*
C_7	*Capacitor*	*0.1μF*
R_1	*Resistor*	*430kΩ*
R_2	*Resistor*	*100kΩ*
C_t	*Tuning capacitor*	*33pF*
